# Records of antibodies in breast milk in postpartum women who have been vaccinated or exposed to COVID-19: A systematic review

**DOI:** 10.12688/f1000research.122237.1

**Published:** 2022-07-13

**Authors:** Eighty Mardiyan Kurniawati, Nur Anisah Rahmawati

**Affiliations:** 1 Department of Obstetrics and Gynecology, Faculty of Medicine, Universitas Airlangga, Surabaya, 60286, Indonesia; 2 Faculty of Public Health, Universitas Airlangga, Surabaya, 60286, Indonesia

**Keywords:** COVID-19, maternal health, neonatal health, human milk, antibody, vaccine

## Abstract

**Background:** Breast milk is a critical element in developing a baby’s immunity through immune transfer. Antibodies are an essential unit of immunity against infection with the SARS-CoV-2 virus. This paper explores antibodies in breast milk in postpartum women who have been vaccinated or exposed to coronavirus disease 2019 (COVID-19). Duration of antibody appearance was studied to determine the adequate time in transferring antibodies by breastfeeding.

**Methods:** Three databases, PubMed, Google Scholar, and ScienceDirect, were used as sources of articles. Inclusion criteria applied in selecting articles were prospective observational study or experimental design study in English, evaluating antibodies in breast milk, and conducted between 2019–2021. Article quality and risk of bias were assessed with Critical Appraisal Skills Programme (CASP). The data found were synthesized in a narrative manner.

**Results:** This systematic review included 20 articles. A total of 306 postpartum women who were infected with COVID-19, 20 postpartum women who had viral symptoms and 495 postpartum women who had been vaccinated were studied. Immunoglobulin A (IgA) and immunoglobulin G (IgG) antibodies were found in the breast milk of infected and vaccinated postpartum women. SARS CoV-2 infection is associated with the presence of IgA dominant, whereas vaccination is related to the presence of IgG dominant. Antibodies persisted from day 10 of onset to 10 months in infected postpartum women and started from three days to six weeks in vaccinated postpartum women. Meta-analysis could not be carried out due to the variety of articles.

**Conclusions:** Antibodies found in breast milk in infected and vaccinated postpartum women have different dominant types. Further research needs to be done regarding the mechanism of antibody transfer in breast milk, longer research duration and studies that directly examine the comparison of antibodies in breast milk in vaccinated and infected postpartum women.

**Registration:** PROSPERO ( CRD42022340859, 23 June 2022).

## Introduction

SARS CoV-2 became a worldwide pandemic and caused changes in the phases of life, one of which was postpartum and neonatal periods. ^1^ Coronavirus disease 2019 (COVID-19) infection during pregnancy, birth and puerperium is associated with a significant increase in morbidity and mortality in mothers and babies. They are considered people susceptible to infection compared to the general population. ^2^ COVID-19-related postpartum deaths have been reported in Brazil, with an estimated 106 deaths in 2020. ^3^ In addition, babies are also vulnerable to COVID-19 infection. The babies born to mothers infected with COVID-19 are more likely to be admitted to the neonatal unit. ^4^ Cases of COVID-19 in children in previous studies were seen to be less frequent, less severe, and the mortality rate very low, but there is growing evidence that they are as susceptible as adults. ^5^ Infants with severe respiratory failure and the prolonged clinical course associated with SARS-CoV-2 exposure may be due to extreme prematurity, immature lungs, and immunocompromised status. ^6^

Breast milk is the only nutrient needed in the first six months of life. ^7^ The best source of nutrition and protection for babies from disease is breast milk. ^8^ Breast milk can be an opportunity and hope for babies to achieve protection. The provision of a COVID-19 vaccine for newborns is not yet available. A recent study found that a COVID-19 vaccination regimen consisting of BNT162b2 was found to be safe, immunogenic, and efficacious in children aged five to 11 years. ^9^ When compared with infants, postpartum mothers have the opportunity to get antibodies through vaccination. The COVID-19 vaccine is an effective way to prevent COVID-19 in breastfeeding people. ^10^ In influenza outbreaks in previous years, influenza vaccines have been shown to increase serum antibodies and reduce disease severity in both mother and baby. Breastfeeding can protect you for at least six months because breast milk contains consistently high levels of actively produced anti-influenza immunoglobulin A (IgA). Infants with fever have fewer episodes of respiratory illness, which implies that breastfeeding may provide local mucosal protection. ^11^ The risk of COVID-19 can be reduced through breastfeeding among children as has been documented for other infections compared to formula feeding. ^12^

The risk of being exposed to the virus and providing nutrition is a dilemma, especially regarding the content of substances contained in breast milk. This is also exacerbated by the rise of fake news and misinformation on social media about the content of the SARS-CoV-2 virus in breast milk, causing breastfeeding issues during the pandemic. ^13^ Mothers will be distraught and ask themselves whether the coronavirus can be transmitted through breast milk and what they can do to protect themselves and their babies. ^8^ The World Health Organization (WHO) said breastfeeding does not need to be stopped during COVID-19 infection or after the mother’s vaccination. ^14^ Research conducted by United Nations Children’s Fund (UNICEF) in five countries in South Asia found that less than 25% of interviewees understood that it was safe to continue breastfeeding. They preferred to give formula milk. ^15^ Meta-analysis conducted in 2020 showed that the SARS-CoV-2 genome is generally not found in the breast milk of individuals infected with COVID-19. ^16^

After the virus content information has been clearly explained, the protection points become an essential part to know. Previous studies have found that there are antibodies in the breast milk of COVID-19-infected mothers, but the duration of the presence of these antibodies is unknown. ^17^ Infected and vaccinated postpartum women allow the formation of an antibody against COVID-19, but the long-term impact on antibody composition and functional activity is unclear. ^18^ Given this context, the purpose of this research is to determine the status of antibodies in breast milk following COVID-19 exposure and vaccination. After finding records of the presence of antibodies, the duration of the appearance of antibodies will also be studied. Knowledge about the duration of antibodies in breast milk determines the adequate time in transferring antibodies by breastfeeding. The current results can be used as a guideline for recommendations to continue breastfeeding and health promotions that emphasize the presence of antibodies in breast milk in vaccinated postpartum mothers.

## Methods

### Study design and search strategy

The status of antibodies in breast milk after exposure to COVID-19 and after vaccination and the duration of antibodies appearing in breast milk were explored by compiling a systematic review. The preparation of this report follows the Preferred Reporting Items for Systematic Review and Meta-Analyses (PRISMA) guidelines. ^19^^,^^39^ This systematic review is registered in PROSPERO ( CRD42022340859, 23 June 2022). Data were collected in July-September 2021. English-language research conducted from 2020 to 2021 according to topics was searched in PubMed (RRID:SCR_004846), Google Scholar (RRID:SCR_008878), and Science Direct. The keywords used were a combination of Medical Subject Heading (MeSH) terms and relevant keywords in a different order: “breast milk”, “breast milk”, “COVID-19”, “antibody”, “immunoglobulin”, “vaccine”, “severe”, “acute respiratory syndrome coronavirus 2”, “COVID19”, “coronavirus disease 2019”, “SARS-CoV-2”. In the articles found, the researcher also examined the literature in the bibliography, including manuscripts that were not captured in the electronic literature search.

### Study selection

The inclusion criteria applied in selecting articles were prospective observational study or experimental design study in English, evaluating antibodies in breast milk, and the study was conducted during the COVID-19 pandemic between 2019 and 2021. The exclusion criteria for this study were case reports, animal studies, letters to editors, study reviews, abstracts without full text. Manuscripts that only discussed breast milk in healthy postpartum mothers were not included.

### Data collection process

Two authors (EMK and NAR) performed title and/or abstract screening independently of the included articles using standard Microsoft Excel (RRID:SCR_016137) forms. The data obtained were combined in one folder and then an assessment was carried out. Each author analyzed all existing manuscripts, and then the results were compared with each other. A third external collaborator was consulted (Hari Paraton) to address disagreements in consensus.

### Data items

The outcomes sought from this study were the presence of antibodies and the duration of the appearance of antibodies in breast milk. The antibodies studied were IgG and IgA against COVID-19. The tool to detect the presence of this antibody used ELISA or a similar working tool. The researcher realized that not all manuscripts had explanations for these two antibody levels, so studies that only discussed one type of antibody were also included in the systematic review. The researcher also realized that not all studies that discussed antibodies also explained the duration of the appearance, so based on the consideration of the limited number of articles, the researcher discussed the data found from existing studies. Unclear information was included in the exclusion criteria.

### Study risk of bias assessment

Researchers conducted a risk of bias assessment study with the help of critical appraisal tools. The Critical Appraisal Skills Programme (CASP) was used to assess the formal article by two independent research team members, EMK and NAR. Questions are designed to help systematically review a cohort study. The use of CASP is based on the 1994 JAMA ‘Users’ guides to the medical literature, which is used for both randomized controlled trials and systematic reviews. This checklist was adapted from Guyatt GH, Sackett DL, and Cook DJ and is used by health care practitioners. ^20^ CASP results are concluded into the category of moderate overall quality and low overall quality. ^21^ The results of the analysis are presented in Table 1.

**Table 1.** CASP checklist for a cohort study.

Author	Did the study address a clearly focused issue?	Was the cohort recruited in an acceptable way?	Was the exposure accurately measured to minimize bias?	Was the outcome accurately measured to minimize bias?	Have the authors identified all important confounding factors?	Have the authors taken account of the confounding factors in the design and/or analysis?	Was the follow up of subjects complete enough?	Was the follow up of subjects long enough?	How precise are the results?	Do you believe the results?	Can the results be applied to the local population?	Do the results of this study fit with other available evidence?	What are the implications of this study for practice?	Overall quality assessment
Juncker *et al.* ^22^	Y	Y	Y	Y	Y	Y	C	C	Y	Y	Y	Y	Y	M
Juncker *et al.* ^40^	Y	Y	Y	Y	N	N	Y	Y	Y	Y	Y	Y	Y	M
Narayanaswamy *et al.* ^41^	Y	Y	Y	Y	Y	Y	C	C	Y	Y	Y	Y	Y	M
Bäuerl *et al.* ^42^	Y	Y	Y	Y	N	N	C	C	Y	Y	Y	Y	Y	M
Duncombe *et al.*, ^43^	Y	Y	Y	Y	N	N	C	C	Y	Y	Y	Y	Y	M
Pace *et al.* ^44^	Y	Y	Y	Y	N	N	C	C	Y	Y	Y	N	Y	M
Fox *et al.* ^45^	Y	Y	Y	Y	N	N	Y	Y	Y	Y	Y	N	Y	M
Demers-Mathieu *et al.* ^46^	Y	Y	Y	Y	N	N	C	C	Y	Y	Y	Y	Y	M
Lechosa-Muñiz *et al.*, ^23^	Y	Y	Y	Y	Y	Y	C	C	Y	Y	Y	Y	Y	M
Gray *et al.* ^25^	Y	Y	Y	Y	Y	Y	Y	Y	Y	Y	Y	Y	Y	M
Low *et al.* ^47^	Y	Y	Y	Y	N	N	Y	Y	Y	Y	Y	Y	Y	M
Jakuszko *et al.* ^48^	Y	Y	Y	Y	N	N	C	C	Y	Y	Y	Y	Y	M
Guida *et al.* ^49^	Y	Y	Y	Y	N	N	C	C	Y	Y	Y	Y	Y	M
Ramìreza *et al.* ^50^	Y	Y	Y	Y	N	N	C	C	Y	Y	Y	Y	Y	M
Perl *et al.* ^51^	Y	Y	Y	Y	N	N	C	C	Y	Y	Y	Y	Y	M
Golan *et al.* ^30^	Y	Y	Y	Y	N	N	C	C	Y	Y	Y	Y	Y	M
Valcarce *et al.* ^52^	Y	Y	Y	Y	Y	Y	C	C	Y	Y	Y	Y	Y	M
Selma-Roy *et al.* ^24^	Y	Y	Y	Y	Y	Y	C	C	Y	Y	Y	Y	Y	M
Juncker *et al.* ^53^	Y	Y	Y	Y	Y	Y	C	C	Y	Y	Y	Y	Y	M
Rosenberg-Friedman *et al.* ^54^	Y	Y	Y	Y	Y	Y	C	C	Y	Y	Y	Y	Y	M

Note: CASP = Critical Appraisal Skills Programme, Y = yes, C = can't tell, N = No, N/A = not applicable, M = moderate overall quality, L = low overall quality.

Further information for the question “What were the results of this study?” has been presented in Table 2 and Table 3.

### Effect measures

Due to the limited number of manuscripts, a meta-analysis could not be completed. The effect that was measured in this study was the number of respondents’ breast milk presentations where antibodies were found in breast milk. The obtained durations were also combined descriptively.

### Data abstraction and synthesis

The study selection process was through a review of the inclusion and exclusion criteria. As suggested in a systematic review of the literature, the analysis in this study was based on the findings of each study. The steps started from extracting the relevant results, sorting, and examining them to identify sub-themes and themes. The type of synthesis used is in the form of narrative synthesis. Narrative synthesis was chosen because it allowed researchers to gather insight into the antibody content in breast milk and we did not perform this meta-analysis. The data are arranged in Tables 2 and 3. Table 2 contains the antibody content in breast milk in mothers exposed to COVID-19 and is written in a systematic table including (1) authors, year, country, (2) research time, (3) study type, (4) COVID-19 confirmation, (5) control group, (6) sampling time, (7) number of infected mothers, (8) antibody test, (9) number of mothers showing antibodies, and (10) duration time. Table 3 contains antibody content in breast milk in mothers vaccinated against COVID-19 and is written in a systematic table including (1) authors, year, country, (2) research time, (3) study type and sample timing, (4) vaccine type, (5) number of vaccinated women and the dose given, (6) antibody test, (7) antibody status, and (10) duration time. Heterogeneity in the data was explored including duration of baseline antibody measurement and population characteristics.

**Table 2.** Characteristics of research on mothers with COVID-19 and antibody status in breast milk.

Authors, year, country	Research time	Study type	COVID-19 confirmation	Control group	Sampling time	No. of infected mothers	Antibody test	No. of mothers showing antibodies, n (%)			Duration time
								IgA	IgG	IgM	
Juncker *et al.*, ^22^ 2021, Netherlands	October 2020 and February 2021	Prospective cohort	PCR	Healthy lactating mother	An average of eight weeks after getting a positive result	165	ELISA	98 (59)	No data available	No data available	10 months after COVID-19 confirmation
Juncker *et al.*, ^40^ 2021, Netherlands	May–August 2020	Longitudinal follow-up	PCR and symptoms	No control used in this research	After the clinical symptoms in 14–143 days	24 confirmed or highly suspected	ELISA	24 (100)	No data available	No data available	Five months (143 days) after the onset of COVID-19 symptoms
Narayanaswamy *et al.*, ^41^ 2021, MMC Worchester, MA	March–September 2020	Prospective cohort	PCR	Bilateral colostrum sample pre-pandemic as control (n = 8)	48 hours of delivery	15	ELISA	11 (73)	11 (73)	5 (33)	No data available
Bäuerl *et al.*, ^42^ 2021, Spain	April–December 2020	Prospective multicenter longitudinal study	PCR and/or after COVID-19 infection	Whole milk pre-pandemic control (n = 13)	Before and after delivery	60	ELISA	51 (72.9)	45 (64.3)	51 (72.9)	206 days after PCR confirmation
Duncombe *et al.*, ^43^ 2021, USA	No data available	Prospective longitudinal cohort study	PCR	PCR negative control (n = 3)	Three weeks to six months post-illness onset	Two	ELISA	2 (100)	2 (100)	1 (50)	10 days to six months after symptom onset
Pace *et al.*, ^44^ 2021, USA	No data available	Prospective study	PCR	Milk sample pre-pandemic control (n = 10)	Average 12.0 ± 8.9 days after onset of symptoms	18	ELISA	14 (76)	15 (80)	No data available	No data available
Fox *et al.*, ^45^ 2020, USA	December 2019	Prospective study	PCR and symptoms	Milk sample pre-pandemic control (n = 10)	14–30 days after symptoms	15	ELISA	12 (80)	2 (13)	1 (7)	No data available
Demers-Mathieu *et al.*, ^46^ 2021, USA	No data available	Unpaired experimental design	PCR	20 with viral symptom control and 16 unexposed	16–84 days	Seven diagnosed	ELISA	26 (96)	26 (96)	26 (96)	No data available

Note: COVID-19 = coronavirus disease 2019, IgA = immunoglobulin A, IgG = immunoglobulin G, IgM = immunoglobulin M.

**Table 3.** Characteristics of research on vaccinated mothers and antibody status in breast milk.

Authors	Year	Country	Research time	Study type and sample timing	Vaccine type	Number of vaccinated women and the dose given	Antibody test	Antibody status	Time
Lechosa-Muñiz *et al.* ^23^	2021	Spain	1 April 2021 to 30 April 2021	Observational study 30 days after the second dose of the vaccine and 30 days after the first dose.	BNT162b2 mRNA-1273 ChAdOx1-S	20 women with a single dose of ChAdOx1-S. 70 women with two doses of BNT162b2, 20 women with two doses of mRNA-1273.	ELISA	Breastfeeding mothers offer their infants IgA and IgG isotype antibodies directed against SARS-CoV-2 protein S in breast milk.	No data available
Gray *et al.* ^25^	2021	No data available	17 December 2020, and 23 February 2021	Prospective cohort study two to six weeks after the second vaccine.	BNT162b2 Pfizer/BioNTech or mRNA-1273 Moderna/National Institutes of Health (NIH)	31 women with two doses of RNA vaccine.	ELISA	The second vaccine dose increased IgG, but not IgA, in maternal blood and breast milk.	No data available
Low *et al.* ^47^	2021	Singapore	No data available	Longitudinal study. Five time points: pre-vaccination (T1), 1–3 days after dose one (T2), 7–10 days after dose 1 (T3), 3–7 days after dose two of COVID-19 mRNA vaccine (T4), and 4–6 weeks after dose two of COVID-19 mRNA vaccine (T5).	BNT162b2 (Pfizer-BioNTech)	14 lactating healthcare workers after the second dose	ELISA	86% (12/14) of the individuals produced SARS-CoV-2-specific IgA, and 100% (14/14) individuals produced SARS-CoV-2-specific IgG in human milk.	86% of individuals produce SARS-CoV-2-specific IgA, and 100% of individuals produce SARS-CoV-2-specific IgG in breast milk within 3-7 days after administration second dose of BNT162b2 vaccine and decreased over time. Significant IgG decreased to 4-6 weeks after the second dose.
Jakuszko *et al.* ^48^	2021	Poland	No data available	Prospective cohort Sampling after the first dose of vaccine, on day 8 ± 1, on day 22 ± 2 (immediately before the second dose), on day 29 ± 3 (which is also day 7 ± 3 after the second dose), and on day 43 ± 4 (which is also day 21 ± 4 after the second dose).	mRNA vaccine BNT162b2 (Pfizer-BioNTech)	32 breastfeeding women.	ELISA	There are IgG and IgA in breast milk. IgG is detectable in serum levels.	Serum and breast milk antibody concentrations were highest on day 29 ± 3 and decreased on day 43 ± 4. The immune response to vaccination was most vital 7 ± 3 days after the second dose of vaccine. Strong secretion of IgA and IgG antibodies in breast milk for six weeks after vaccination.
Guida *et al.* ^49^	2021	Italy	No data available	Cohort prospective observational study, 17 breastfeeding women and 10 volunteers 20 days after the first dose and seven volunteers seven days after the second dose.	mRNA BNT162b2 vaccine	10 women after the first dose, seven women after the second dose.	ELISA	Anti-SARS CoV-2 S-IgA, IgG, and IgM antibodies in the breast milk of vaccinated women.	7 days after the second dose.
Ramìreza *et al.* ^50^	2021	No data available	22 February to 4 April 2021	Prospective cohort, 14 days after their second dose of vaccine.	94%, BNT162b2 mRNA COVID-19 vaccine and 6% COVID-19 mRNA-1273 vaccine	98 vaccinated, and the 24 control participants.	The SARS-CoV-2 IgG Architect Abbott®	All participants had IgG antibodies, and 89% of them had IgA antibodies against SARS-CoV-2 in breast milk.	No data available
Perl *et al.* ^51^	2021	Israel	December 2020 and January 2021	Prospective cohort study before administration of the vaccine and then once weekly for six weeks starting at week two after the first dose.	All participants received two doses of the Pfizer-BioNTech	44 breastfeeding women after the first dose.	ELISA	Strong neutralizing effects, protective effect against infection in the infant due to SARS-CoV-2.	SARS-CoV-2-specific IgA and IgG antibodies in breast milk six weeks after vaccination. IgA secretion was early, followed by a spike in IgG after four weeks (a week after the second vaccine).
Golan *et al.* ^30^	2021	USA	December 2020 to June 2021	Prospective cohort, 50 breastfeeding women collected from a subset of infants whose mothers received the vaccine during lactation or pregnancy (4–15 weeks after mothers’ second dose).	mRNA46-based vaccines for COVID-19 (mRNA-1273 and BNT162b2)	50 breastfeeding mothers after second dose.	ELISA	Anti-SARS-CoV-2 IgG and IgM levels significantly increased in maternal plasma, and significant transfer of anti-SARS-CoV-2 IgA and IgG antibodies to milk. Anti-SARS-CoV-2 IgG antibodies were not detected in the plasma of infants whose mothers were vaccinated during lactation.	No data available
Valcarce *et al.* ^52^	2021	USA	December 2020 to March 2021	Prospective observational study, 22 lactating health care workers. There were three time points (pre-vaccination, post-first vaccine, and post-second vaccine doses).	SARS-CoV-2 mRNA vaccine (Pfizer-BioNTech or Moderna)	20 lactating health care mothers.	ELISA	Significant secretion of SARS-CoV-2-specific IgA and IgG in human milk and plasma is discovered after SARS-CoV-2 vaccination.	No data available
Selma-Roy *et al.* ^24^	2021	Spain	January to April 2021	Prospective cohort observational study, 75 lactating women. Seven time points were collected from baseline up to 25 days after the 1st dose, and same points were collected for mRNA vaccines 30 days after second dose.	mRNA vaccines (BNT162b2 and mRNA-1273) and adenovirus-vectored vaccine (ChAdOx1 nCoV-19)	75 lactating women.	ELISA	High intervariability for IgA antibodies. IgG levels were significantly higher than those observed in milk from women who had COVID-19, while IgA levels were lower.	No data available
Juncker *et al.* ^53^	2021	Netherlands	2021	Prospective longitudinal study, 16 samples of human milk were collected according to a schedule: one sample before the first vaccination and one sample on days 3, 5, 7, 9, 11, 13, and 15–17 days after the first vaccination. This schedule was the same for the second vaccination.	mRNA based BNT162b2 vaccine	20 women after the second dose, six women after the first dose.	ELISA	A SARS-CoV-2-specific antibody IgA response was observed in human milk.	IgA starting to increase between days 5–7 after the first dose and declining after day 15, on average.
Rosenberg-Friedman *et al.* ^54^	2021	No data available	2021	Prospective cohort study approximately five months postpartum in the first dose (mean 154 days, range 68−382) and the second dose 21 days later.	BNT162b2 mRNA vaccination	10 lactating health care providers in first dose and second dose.	ELISA	IgG and IgA with neutralization capacity.	14 days after the second dose.

Note: COVID-19 = coronavirus disease 2019, IgA = immunoglobulin A, IgG = immunoglobulin G, IgM = immunoglobulin M.

### Reporting bias assessment

The researcher was unable to review the funnel plot in this case due to the limitations of the manuscript so that the risk assessment of bias from missing results in the synthesis was carried out using an emerging risk approach and was reported as a research constraint.

### Certainty assessment

The main domain used to assess the certainty of evidence was the risk of bias through critical appraisal and the inconsistency of the results of the research included in the review.

## Results

### Results of article screening

**Figure 1.**
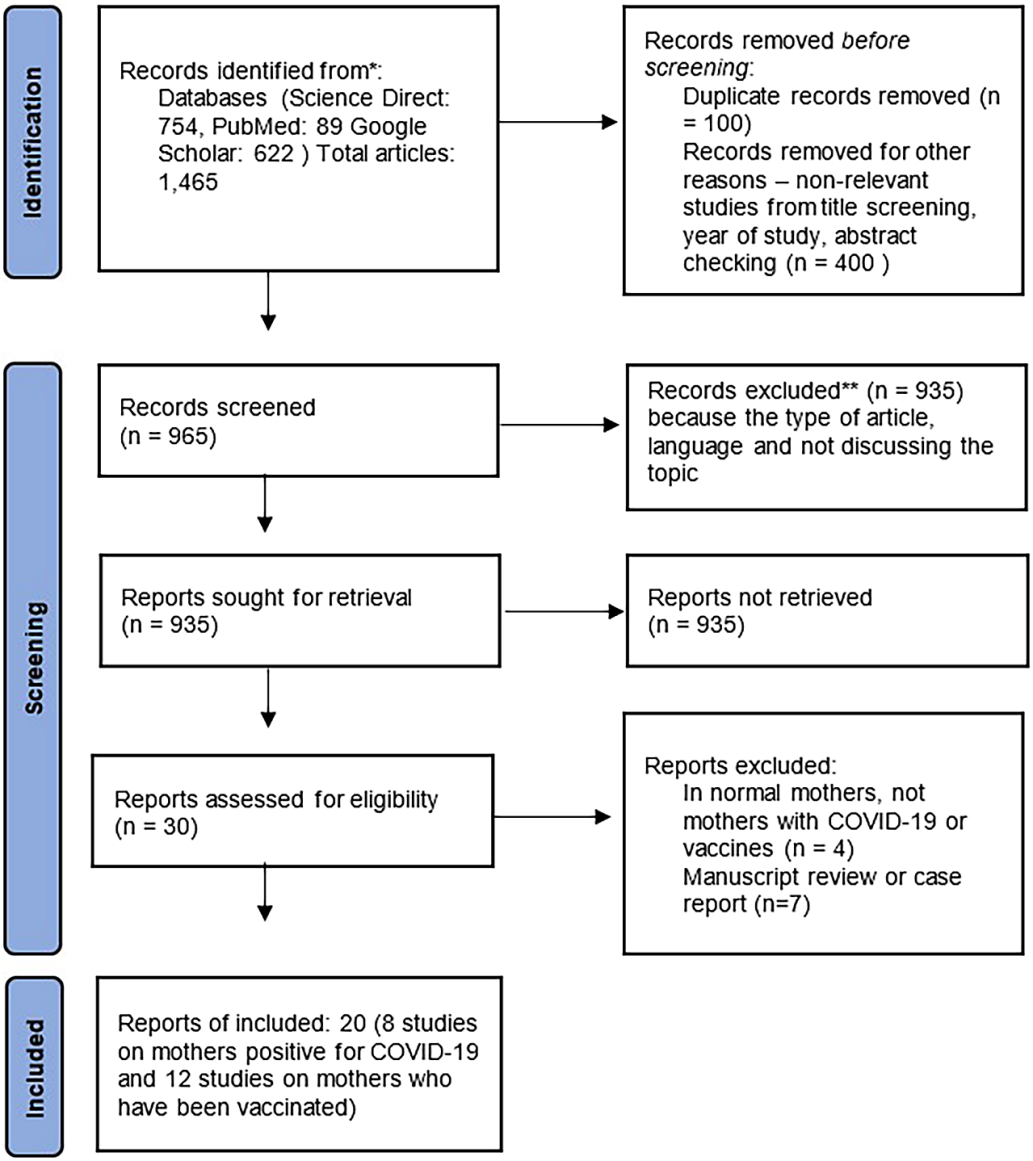
Flowchart study selection. COVID-19, coronavirus disease 2019.

In searching the database, 1,465 abstracts were found from searches with relevant keywords. The authors then screened results for possible inclusion. The findings of 935 articles were excluded from the eligibility list. The articles did not meet the criteria, including the type of manuscript, the language used, and the topics discussed under the proposed title. After this stage, the researcher tried to re-examine the assessment results, but the manuscript finally entered the exclusion criteria. After screening, 30 full-text articles were selected and examined in detail to determine eligibility. Furthermore, 20 articles were determined that met the requirements. There were eight studies on the antibody status of infected breastfeeding mothers and 12 studies on antibody status of vaccinated breastfeeding mothers included. Figure 1 shows the study selection flowchart. The quality of the research is in the moderate category and is arranged in Table 1.

### Antibody status in breast milk from infected mother

A total of 306 breastfeeding mothers who were positive for COVID-19 and 20 of them who had viral symptoms and had a very high likelihood that they had been exposed to COVID-19 were studied to determine antibody status in breast milk. Samples were taken within 48 hours after delivery to six months after infection. Table 2 describes antibody status in mothers who had been infected with COVID-19. The samples included in this review are breastfeeding mothers with positive PCR confirmation and mothers who had a very high likelihood that they had been exposed to COVID-19 when viewed from the symptoms they had. Almost all studies used controls, namely comparisons with healthy breastfeeding mothers and milk samples taken during the pre-pandemic period. One study did not include controls, namely a longitudinal study. Detection of all antibodies was performed using ELISA. Most studies found that breast milk contains IgA, immunoglobulin G (IgG), and immunoglobulin M (IgM). IgA was the dominant antibody found, while IgG and IgM were not always found in individuals. However, infected mothers also did not always have IgA antibodies against COVaID-19, which was found in a study by Juncker *et al.* (2021), who found that 59% of mothers had these antibodies. ^22^

### Antibody status in breast milk from vaccinated mother

The characteristics of the study on antibody status in the breast milk of vaccinated mothers can be seen in Table 3. A total of 495 breastfeeding mothers who had been vaccinated, received the first and second doses of various types of vaccines and had never had COVID-19 were analyzed. IgG and IgA antibodies were found in the breast milk of vaccinated mothers. No studies showed IgM data. Administration of the second dose of the vaccine was associated with an increase in IgG in breast milk. Despite evidence of increased antibodies, anti-SARS-CoV-2 IgG antibodies were not detected in the plasma of infants whose mothers were vaccinated during breastfeeding. IgG levels were higher than IgA levels in the breast milk of vaccinated mothers.

### Time duration of antibodies appearing

The duration of antibody time associated with PCR confirmation or symptoms, breast milk testing, and vaccine administration is shown in Figure 1. In mothers infected with COVID-19, the appearance of antibodies was detected on day 10 and could persist for up to 10 months. IgA levels are known to decrease over time, while IgG is relatively stable. In vaccinated mothers, antibody status differed between mothers vaccinated with dose one and dose two. Most studies found the presence of antibodies after the first dose, although in small amounts. Early appearance could be detected three days after vaccination up to six weeks in both dose one and dose two of vaccination. The highest levels and immune response were found about four weeks after vaccination, but at the second dose, the antibody appeared between days four and 10. After that, there may be a chance to experience a decline before the sixth week, especially on day 15 or day 43 ± 4. These studies stopped examining antibodies at the sixth week. Therefore, the data provided are limited to detection at the sixth week. The results of existing studies indicate that it is not known whether, after six weeks, there is still a decrease or whether it persists at a certain point.

### Comparison of antibody levels by type of vaccine

Not all studies discussed the comparison of antibodies in various types of vaccines. There were two studies that explored this. Research conducted by Lechosa-Muñiz *et al.* (2021) concluded that BNT162b2 or mRNA-1273 vaccinated mothers had higher levels of IgG antibodies compared to ChAdOx1-S (p < 0.001 and p = 0.001), respectively. ^23^ Mean values in serum from mothers vaccinated with BNT162b2 or mRNA-1273 *vs.* ChAdOx1-S (0.12, 0.16, and 0.02, respectively; p < 0.001) on IgA antibodies. Regardless of the commercial vaccine administered, all vaccinated breastfeeding mothers had serum anti-S1 IgG antibodies in response to vaccination against SARS-CoV-2. ^23^ Research conducted by Selma-Royo *et al.* (2021) found that mothers vaccinated with Moderna and BioNTech/Pfizer after the first dose had a higher increase in breast milk anti-SARS-CoV-2 IgG than Oxford/AstraZeneca (p < 0.0001). ^24^ Anti-SARS-CoV-2 IgA levels were higher in Moderna than in the Oxford/AstraZeneca (p < 0.0001) and BioNTech/Pfizer groups (p = 0.002). After the second dose, no difference was observed between the two mRNA-based vaccines. Moreover, after the second dose, IgG levels reached a higher level than the first dose, whereas IgA levels did not increase further. ^24^ Gray *et al.* (2021) found that a booster dose of the vaccine increases SARS CoV-2 IgG, but not IgA, in maternal blood and breast milk. ^25^

## Discussion

The antibody status in the breast milk of mothers who have been exposed to COVID-19 or are highly suspected and those who have been vaccinated against COVID-19 differs. The dominant antibody in mothers infected with COVID-19 is IgA, while the predominant antibody in vaccinated mothers is IgG, according to Young *et al.* (2021). ^18^ Vaccination is associated with an increase in dominant IgG and IgA antibodies after direct viral infection. This varies greatly. Breast milk from both groups showed neutralizing activity against live SARS-CoV-2 virus, which could be attributed to SARS-CoV-2 IgA and IgG antibodies. ^18^ The dominant IgA response in breast milk can be found according to previous studies, namely the result of natural infection. ^26^

Antibody transfer through breast milk is an evolutionary strategy for enhancing immunity early in life. ^27^ Research conducted by Pullen *et al.* (2021) in which they applied a serologic system to characterize SARS-CoV-2 specific antibodies in maternal serum and breast milk found a preferential transfer of antibodies capable of causing neutrophil phagocytosis and neutralization. Distinct SARS-CoV-2-specific antibody response was observed in serum and breast milk from nursing individuals previously infected with SARS-CoV-2, with a predominant transfer of IgA and IgM into breast milk. ^27^ IgA is the most essential class of Ig provided by breast milk to infants because it acts in the intestines while the secretory IgA (SIgA) produced by infants is still in development. ^28^ The presence of IgM in some samples suggests the possibility that breast milk may have a protective effect on the newborn. ^29^

Although IgGs are present in breast milk, they are functionally attenuated. ^27^ Emerging data from vaccinated pregnant and lactating women suggest that vaccine-induced transfer may be altered due to unusually high levels of IgG antibody induced by mRNA vaccines approved by the current Emergency Use Authorization. ^25^ It gives the baby strong IgA and IgG antibodies and may increase immunity compared to natural infection. ^27^ Nevertheless, the IgG transfer scheme still needs to be studied further because different studies can find different results. A study conducted by Golan *et al*. (2021) found that no IgG was found in the plasma of infants whose mothers were vaccinated during lactation. ^30^ Although high levels of IgG were found in breast milk, these antibodies may not be transferred effectively to the baby. IgA antibodies produced after vaccination with the Pfizer/BioNTech vaccine resist the gastric phase but are degraded during the intestinal phase of the infant’s digestion. By contrast, IgG is more susceptible to degradation in both digestive phases. ^31^ The results of another study found that maternal SARS-CoV-2 IgG was efficiently transferred across the placenta when infection occurs more than two months before delivery. Passive immunity inherited from the mother can last in infants for up to six months. Neonates can mount a strong antibody response to perinatal SARS-CoV-2 infection. ^32^ Antibodies shown from breastfeeding may have a protective effect on the recipient infant, provided that the infant has not increased its immune response to infection. ^33^

Studies showing the duration of antibody persistence in breast milk are limited. Antibodies can be detected in mothers who are breastfeeding and infected with COVID-19 as early as day 10 and can last for up to 10 months. IgA levels are known to decrease over time, while IgG is relatively stable. In lactating mothers who were vaccinated, the trial was only up to six weeks in duration. Antibodies have been detected after the first dose of vaccination for three days and can last up to six weeks. Before six weeks, it can decrease. It is not known whether the antibody status will still decrease or persist at a certain point. Individual research by Young *et al.* (2021) showed that IgG began to decline by 90 days after the second vaccine dose. ^18^ This suggests that further research is needed to investigate the exact duration of antibodies in breast milk after vaccination.

The type of vaccine is related to the number of antibodies detected in breast milk even though IgG status is more dominant than IgA and IgM. Post-vaccination IgG levels reached levels similar to those of terminally ill COVID-19 patients and demonstrated a decreased breadth of antibody responses targeting the endemic coronavirus. ^34^ Another study conducted by Dashdorj *et al.* (2021) found that responses from high to low were Pfizer/BioNTech, AstraZeneca, Sputnik V and Sinopharm, respectively. ^35^ These different responses also allow different amounts of antibodies to be transferred into breast milk.

Based on the results of the opportunity for antibody transfer, breastfeeding is highly recommended for mothers infected with COVID-19 and mothers who have been vaccinated. Research conducted by Verd *et al.* (2021) found that in a sample of children visiting emergency services with potential symptoms of COVID-19, a higher prevalence of positive SARS-CoV-2 reverse transcriptase (RT)-PCR test results among those who were formula-fed exclusively compared to those who have been breastfed. Breastfeeding can reduce the risk of exposure to COVID-19 and other infections in children, compared to formula feeding. ^12^ It is vital to continue to breastfeed according to the available time duration. Therefore, women with confirmed COVID-19 must adhere to standard precautions for contact with breastfeeding. ^36^ The need to encourage breastfeeding may be justified, at least during the first six months of life, when the infant’s secretory IgA production is insignificant. ^37^ Apart from direct breastfeeding, every mother has the right to choose to breastfeed her baby and health workers need to help ensure good handwashing practices before and after expressing the milk. ^38^

### Limitations and recommendations

Most of these studies only involve a small number of samples, and they did not find a mechanism for antibody transfer during breastfeeding. We recommend that future research explores antibody testing with a more extended research duration (more than six weeks) in vaccinated postpartum women. In order to ensure that the reported time is more accurate, multiple individual studies that directly discuss the comparison of antibodies in vaccinated mothers with mothers exposed to COVID-19 and the assessment of antibody transfer mechanism by breastfeeding may be useful. In addition, the researcher was unable to compile a meta-analysis because it included various types of academic papers.

The strength of this study is that there have not been many studies that have reviewed the comparison of antibody types and duration of antibodies in infected and vaccinated postpartum women. The results obtained can support evidence-based health promotion to support continued breastfeeding during the pandemic and allow for the right time to support antibody transfer.

## Conclusions

IgA and IgG antibodies were found in the breast milk of infected and vaccinated postpartum women. Infection with SARS CoV-2 is related with the presence of IgA, but vaccination is associated with increased IgG. Antibody levels persisted from day 10 of onset to 10 months in infected breastfeeding mothers and start from three days to six weeks in vaccinated breastfeeding mothers. Antibodies produced in breast milk in infected and vaccinated postpartum women have different dominant types. Further research needs to be done regarding the mechanism of antibody transfer in breast milk, longer research duration and studies that directly examine the comparison of antibodies in breast milk in vaccinated and infected postpartum women.

## Data availability

### Underlying data

All data underlying the results are available as part of the article and no additional source data are required.

## Reporting guidelines

Zenodo: PRISMA checklist for ‘Records of antibodies in breast milk in postpartum women who have been vaccinated or exposed to COVID-19: A systematic review. https://doi.org/10.5281/zenodo.6785317. ^39^

Data are available under the terms of the Creative Commons Attribution 4.0 International license (CC-BY 4.0).
