# Supplementary material for: Records of antibodies in breast milk in postpartum women who have been vaccinated or exposed to COVID-19: A systematic review
Source: F1000Res. 2024 Jul 12;11:785. Originally published 2022 Jul 13. [Version 4] doi: 10.12688/f1000research.122237.4 (PMC11249528; doi:10.12688/f1000research.122237.4)
Supplement: Peer review history

# Reviewer report: Reviewer response for version 4

**DOI:** 10.5256/f1000research.168920.r302313

**Published:** 2024-07-15

**Authors:** Guglielmo Stabile

**Affiliations:** 1 Department of Obstetrics and Gynaecology, Institute for Maternal and Child Health-IRCCS “Burlo Garofolo”, Trieste, Italy; 2 Gynecology and Obstetrics, Universita degli Studi di Foggia (Ringgold ID: 18972), Foggia, Apulia, Italy

The manuscript is suitable for indexing.

Are the rationale for, and objectives of, the Systematic Review clearly stated?

Yes

Is the statistical analysis and its interpretation appropriate?

Yes

Are sufficient details of the methods and analysis provided to allow replication by others?

Yes

Are the conclusions drawn adequately supported by the results presented in the review?

No

Reviewer Expertise:

Obstetrics and Gynecology

I confirm that I have read this submission and believe that I have an appropriate level of expertise to confirm that it is of an acceptable scientific standard.

# Reviewer report: Reviewer response for version 3

**DOI:** 10.5256/f1000research.138773.r243406

**Published:** 2024-02-27

**Authors:** Guglielmo Stabile

**Affiliations:** 1 Department of Obstetrics and Gynaecology, Institute for Maternal and Child Health-IRCCS “Burlo Garofolo”, Trieste, Italy; 2 Gynecology and Obstetrics, Universita degli Studi di Foggia (Ringgold ID: 18972), Foggia, Apulia, Italy

Dear Authors, 

 Thank you for allowing me to review this interesting manuscript. This manuscript is a systematic review on the presence, type and duration of antibodies in vaccinated and post infected breastfeeding mothers. 20 articles met the criteria for the authors,8 studies of infected breastfeeding mothers and 12 on antibodies of vaccinated breastfeeding mothers. The article is well written. The subject is of global interest but in my opinion the systematic review should be revised. Furthermore the discussion section and conclusions should be expanded. In fact, In vaccinated women the different sampling times could justify the absence of Ig A in some patients’ milk and serum samples and this should be analysed in the manuscript. The differences in antibody concentrations are directly related to the time elapsed between sampling and administration of the vaccine. The faster decline of IgA could be related to IgA’s natural kinetics. A correlation between milk and blood antibody concentrations should be analyzed or cited. 

 For this scope I suggest reading and cite these articles:  ref [1], [2]

 These articles could led to a more complete review and discussion

Are the rationale for, and objectives of, the Systematic Review clearly stated?

Yes

Is the statistical analysis and its interpretation appropriate?

Yes

Are sufficient details of the methods and analysis provided to allow replication by others?

Yes

Are the conclusions drawn adequately supported by the results presented in the review?

No

Reviewer Expertise:

Obstetrics and Gynecology

I confirm that I have read this submission and believe that I have an appropriate level of expertise to confirm that it is of an acceptable scientific standard, however I have significant reservations, as outlined above.

## References

[1] : IgG and IgA Antibodies Post SARS-CoV-2 Vaccine in the Breast Milk and Sera of Breastfeeding Women. *Vaccines (Basel)* .2022;10(1) : 10.3390/vaccines10010125 10.3390/vaccines10010125 35062786 PMC8778843

[2] : Quantification of Specific Antibodies Against SARS-CoV-2 in Breast Milk of Lactating Women Vaccinated With an mRNA Vaccine. *JAMA Netw Open* .2021;4(8) : 10.1001/jamanetworkopen.2021.20575 e2120575 10.1001/jamanetworkopen.2021.20575 34379127 PMC8358734

# Response

**Published:** 2024-07-10

**Authors:** eighty mardiyan kurniawati

**Affiliations:** Airlangga University, Indonesia

Dear Reviewer,

 Thank you for the suggestion about our article. We have revised the version and we hope this is suitable for your request. There is an addition to the discussion regarding the effect of post-vaccination examination time on antibody levels. We also add a discussion on the relationship between antibodies in the blood and human milk. We also cited the article that you mentioned.

 Thank you. We hope the article's status will change to be approved. 

 Kind regards

# Reviewer report: Reviewer response for version 3

**DOI:** 10.5256/f1000research.138773.r151059

**Published:** 2022-09-28

**Authors:** Nicole Cacho

**Affiliations:** 1 Department of Pediatrics, Division of Neonatology, University of Florida, Gainesville, FL, USA

Authors Kurniawati and Rahmawati revised the manuscript as requested. Thank you for the opportunity to review this important work.

Are the rationale for, and objectives of, the Systematic Review clearly stated?

Yes

Is the statistical analysis and its interpretation appropriate?

No

Are sufficient details of the methods and analysis provided to allow replication by others?

Partly

Are the conclusions drawn adequately supported by the results presented in the review?

Partly

Reviewer Expertise:

Breast milk and lactation research in the NICU

I confirm that I have read this submission and believe that I have an appropriate level of expertise to confirm that it is of an acceptable scientific standard.

# Reviewer report: Reviewer response for version 2

**DOI:** 10.5256/f1000research.137772.r148869

**Published:** 2022-09-15

**Authors:** Nicole Cacho

**Affiliations:** 1 Department of Pediatrics, Division of Neonatology, University of Florida, Gainesville, FL, USA

Thank you for the opportunity to review this revised version of the manuscript.

 Authors Kurniawati and Rahmawati revised the original manuscript “Records of antibodies in breast milk in postpartum women who have been vaccinated or exposed to COVID-19: A systematic review.” In short, this study explored the potential immune protection provided in breast milk by examining 20 studies that focused on breast milk SARS-CoV-2 antibodies.  The revised manuscript is now more comprehensive and will add to the literature on post infection and vaccination breast milk antibodies.  There are a few points to be addressed prior to indexing.

1. Valcarce et al. did compare the levels of breast milk antibodies after vaccination of two separate vaccine types. The data can be found at the bottom of Supplementary Table 1. 
2. In the manuscript when discussing antibodies, it’s important to distinguish the type of antibody (serum vs. breast milk).  For instance, in the section “Comparison of antibody levels by type of vaccine”  there are three separate lines where the type of antibody is unclear. See lines 3, 8 and 11. 

After these points are addressed, I would recommend the manuscript for indexing.

Are the rationale for, and objectives of, the Systematic Review clearly stated?

Yes

Is the statistical analysis and its interpretation appropriate?

No

Are sufficient details of the methods and analysis provided to allow replication by others?

Partly

Are the conclusions drawn adequately supported by the results presented in the review?

Partly

Reviewer Expertise:

NA

I confirm that I have read this submission and believe that I have an appropriate level of expertise to confirm that it is of an acceptable scientific standard, however I have significant reservations, as outlined above.

# Response

**Published:** 2022-09-17

**Authors:** eighty mardiyan kurniawati

**Affiliations:** Airlangga University, Indonesia

Dear Reviewer

 Thank you for providing additional suggestions to improve this manuscript. We have revised the paragraph in the 'comparison of antibody levels by type of vaccine' section. Hopefully our revision is in accordance with the suggestions you give and can be approved. Thank you for your time.

 Best regards

 Eighty M.K

# Reviewer report: Reviewer response for version 1

**DOI:** 10.5256/f1000research.134202.r144205

**Published:** 2022-08-18

**Authors:** Nicole Cacho

**Affiliations:** 1 Department of Pediatrics, Division of Neonatology, University of Florida, Gainesville, FL, USA

Kurniawati and Rahmawati’s interesting manuscript “Records of antibodies in breast milk in postpartum women who have been vaccinated or exposed to COVID-19: A systematic review” summarizes the various manuscripts on breast milk antibodies following COVID-19 exposures and vaccinations. It is obvious the authors worked hard to review the manuscripts and compose the manuscript.

 The article highlighted the specific antibody findings of trends of which type of antibodies were more prevalent after vaccination compared to COVID-19 infection. This extensive work on reviewing 20 cohort studies has highlighted trends in antibodies and laid groundwork for future systematic reviews on studies with longer follow up and possible meta-analysis. Unfortunately, the differences in methods and lack of homogeneity made it impossible to perform a meta-analysis. This work is an important addition to the literature on breast milk immunity in the setting of a global pandemic with COVID-19.

 Major Revisions include the following:

1. Overall, the grammar needs extensive editing.
  This is especially needed in the Introduction section. Many of the sentences below are confusing to the extent that I unsure of the meaning the author is trying to convey.
  Introduction section with confusing sentences (page numbers refer to the pdf version of the manuscript):
  Page 1, 1 ^st^ paragraph, First line: …and caused changes in the phases of life.  In what ways?
  Page 1, 1 ^st^ paragraph, 3 ^rd^ sentence: Consider changing to New mothers and their infants are more susceptible to infection compared to the general population.
  Page 1, 2 ^nd^ paragraph, “Breast milk is the only nutrient needed in the first 6 months of life.”  Did you mean only nutrition?
  Page 1, 2 ^nd^ paragraph, “Breast milk can be an opportunity and hope for babies to achieve protection”.  Would take out hope to make it more scientific. 
  Page 1, 2 ^nd^ paragraph, “Breastfeeding can protect “you” for at least six months because breast milk contains…” Vague you is confusing. Instead would say “Breastfeeding can provide protection for at least the first 6 months because breast milk contains…
  Page 1, 3rd paragraph, The risk of being exposed to the virus and providing nutrition is a dilemma…  What does this mean?  Dilemma to whom? To the mom, or for the baby? Why? 
  Page 1, 3 ^rd^ paragraph, would not use “fake news” which is slang, instead keep the word misinformation. 
  Discussion section sentences which need clarification
  Page 11, 1 ^st^ discussion paragraph, “This varies greatly”, What varies greatly?
  Page 11, 1 ^st^ discussion paragraph, “Breast milk from both groups showed neutralizing activity…” Which groups?
  Page 11, 2 ^nd^ discussion paragraph, “IgA is the most essential glass of Ig provided by breast milk…produced by infants is still in development.” What is still in development?
  "The presence of IgM in some samples (breast milk) suggests the possibility that breast milk may have a protective effect on the newborn.” In what way in the context of this systematic review?
  Page 11, 3 ^rd^ paragraph, 3 ^rd^ sentence, “It gives the baby strong IgA and IgG antibodies and may increase immunity comparted to natural infection.” What is “it”?  mRNA vaccines?  If so, say these vaccines or mRNA vaccines. 
  Page 12 of discussion first sentence, “Needs to be studied further because different studies can find different results.” This sentence is too vague.  Do you mean that studies report conflicting results?  If so, say that. 
  Page 12, 3 ^rd^ paragraph , Dashdorj et al. 2021, which antibody?
2. Be consistent throughout the manuscript and tables when describing the population.
  Determining the difference between exposed to COVID-19 or infected with COVID-19 (or even suspected infection with COVID-19). This point is important as many people that are exposed may not know they are exposed and if asymptomatic will likely not get tested. Thus, simply exposed does not seem like the description that describes the majority of the population in these 20 studies. Be consistent in the terms of infected or exposed. For example, the title of Table 2 is “Characteristics of research on mothers with COVID-19 and antibody status in breast milk” but on Page 4 under the section Data abstraction and synthesis, “Table 2 is antibody contact in mothers exposed to COVID-19” but later on Page 10 under Antibody status in breast milk from infected other.“ Table 2 disuses antibody status in mothers who had been infected with COVID-19. Based on the majority of the studies confirming COVID-19 with PCR (with the exception of 1 study by Bauerl et al. 2021 which had CPR and/or after COVID-19 infection), I would advise changing the title of Table 2 to “Characteristics of research on mothers infected with COVID-19 and antibody status in breast milk”. Furthermore, I would not refer to Table 2 in the text as mothers who had been exposed. 
3. Recommend removing or modifying certain points that are not necessary to the flow of the manuscript. 
  Page 1, 2 ^nd^ paragraph, “Infants with fever have fever episodes of respiratory illness, which implies that breastfeeding may provide local mucosal protection?" I’m not sure what that means. Does this mean all infants with fever, or with COVID-19? If it’s a general statement of all infants with fever of unknown cause, it does not add to the paragraph. If it’s meaning infants with COVID-19 with fever, then say that.
  Page 12, 4 ^th^ paragraph, I would remove the sentence “Therefore, women with confirmed COVID-19 must adhere to standard precautions for contact with breastfeeding." Contact precautions were not previously discussed and may be confusing or send unclear messages. If it is strongly desired to keep this sentence, would change must to “should be advised” and also cite WHO, CDC and AAP recommendations for precautions.
  Page 12, 4 ^th^ paragraph, I would update the sentence “The need to encourage breastfeeding may be justified at least during the first 6 moths of life, when the infant’s secretory IgA production is insignificant.” There are clear and updated recommendations on the duration of breastfeeding from American Academy of Pediatrics, UNICEF and WHO: which all say exclusive breastfeeding for first 6 months and then up to 2 years of age. You could then highlight the importance of the first 6 month based on infant’s low production of sIgA after discussion the general breastfeeding duration recommendations. 
  WHO and UNICEF recommend:
  - early initiation of breastfeeding within 1 hour of birth;
  - exclusive breastfeeding for the first 6 months of life; and
  - introduction of nutritionally-adequate and safe complementary (solid) foods at 6 months together with continued breastfeeding up to 2 years of age or beyond.
  (9 June 2021)
https://www.who.int/news-room/fact-sheets/detail/infant-and-young-child-feeding
  AAP supports continued breastfeeding, along with appropriate complementary foods introduced at about 6 months, as long as mutually desired by mother and child for 2 years or beyond. These recommendations are consistent with those of the World Health Organization (WHO).  (See below for citation)
  Joan Younger Meek, Lawrence Noble, Section on Breastfeeding; Policy Statement: Breastfeeding and the Use of Human Milk. *Pediatrics* July 2022; 150 (1): e2022057988. 10.1542/peds.2022-057988
4. Important to use language that qualifies interpretations of studies that suggest findings but not strongly. For instance, on Page 12, 4 ^th^ paragraph, “Breastfeeding can reduce the risk of exposure to COVID-19 and other infections in children when compared to formula feeding (reference 12 by Verd et al. 2021)." This is too strong of a statement since the cohort was retrospectively reviewed in a secondary analysis and used “Any breastfeeding” vs. exclusive formula feeding and these were in children up to 13 years of age. The conclusion section uses the word suggest that there is protection with breastfeeding. Thus, I recommend changing the work “can” reduce to “may” reduce. 
5. Important point to consider - Mechanism of antibody transfer in the breast milk. I am uncertain what the meaning of this statement is. As per World Health Organization, the presence of IgA in breast milk is one of the ways in which breastfeeding protects infants against infection and death. This appears to be a big part of the conclusion section for future direction but it is not clear that it is based on the results presented. 
6. Tables
  Table 1: The question of How precise are the results? Is not a yes/ no question. Per CASP checklist for cohort study the included HINT was Look for range of confidence interval. I would also include p values if given. If not available, list “no data available” for that particular study. This is important for the statistical analysis portion. After you can compare and contrast studies within each group of vaccinated or infected mothers. 
  Table 2: To make it more consistent for no. of infected mothers, just include the numerical value, (two are in words).
  Table 2: Bauerl et al. sampling time is listed as before and after. How many days or weeks? Also how is infection defined in this study, if not by PCR?
  Table 2: last entry Demers-Mathieu et al lists sampling time as 16-84 days. What is that in relation to?
  Table 2 Heading Duration time: What is this the duration of? Follow up with patients?
  Table 3 Heading Time: of what? Timing after vaccination when antibody levels are measured?
  Table 3: Perl et al: which antibodies under “Antibody status”?
  It would be informative to see an addition of antibody breakdown by type added to Table 3. 
7. Figures
  Figure 1 is the Flow chart of the study selection but Figure 1 is mentioned under Results subheading Time duration of antibodies appearing and is described as showing “Duration of antibody time associated with PCR confirmation or symptoms, breast milk testing, and vaccine administration. Was this supposed to be labeled as Figure 2.  If so, I didn’t see a Figure 2.
  Figure 1 Flowchart is confusing because the numbers do not add up as they are explained.  It appears that from the 1465, 500 were eliminated to leave 965. Then another 935 were eliminated to leave 30. Next 10 were eliminated to ultimately leave 20 studies.  Each elimination should be explained clearly and the numbers should match up. Minor point: From 30 to 20 in the last 2 boxes in the flowchart reports excluded explained 11 that were eliminated but 30-11=19, not 20. Please revise this flowchart using an example from another systematic review. 

**Small revision points:**

 In methods on keyword search, it seems that “breast milk” and “COVID-19” were typed twice as an error.  Please fix this. These are details of the methods and analysis to allow replication by others.

 What is meant by “unclear information was included in the exclusion criteria”? Is this for a few of the studies that were not included or is this describing the majority of the 20 studies that were included?

 What is a funnel plot? If you mention it, you may want to explain it. 

 Page 10, last sentence, typo: COVaID-19 instead of COVID-19.

 Page 11, Results section, Heading Antibody status in breast milk from vaccinated, second to last sentence, Serum levels in infants appears to only be included in one study, so please provide the reference for that study as a superscript and also mention it was only one of the 20 studies. Also, how many infants had serum levels? Not many mothers would want you to poke their babies to get a blood draw.  

 Page 11, Results section, heading time duration of antibodies appearing, Last sentence is an interpretation of results thus, belongs in discussion section (which you already included on page 12, 2 ^nd^ paragraph), so you can delete this sentence. 

 Limitations and recommendations section: instead of the last sentence, “…because it included various types of academic papers.” Consider saying lack of homogeneity. 

 Why do Pace et al. and Fox et al. not fit with other available evidence? This should be explained in the discussion section.  Personally, from Table 2 the Demers-Mathieu et all paper has all 26 patients with all antibodies in milk including IgA, IgG and IgM. I don’t believe those results. Do you?

Are the rationale for, and objectives of, the Systematic Review clearly stated?

Yes

Is the statistical analysis and its interpretation appropriate?

No

Are sufficient details of the methods and analysis provided to allow replication by others?

Partly

Are the conclusions drawn adequately supported by the results presented in the review?

Partly

Reviewer Expertise:

NA

I confirm that I have read this submission and believe that I have an appropriate level of expertise to confirm that it is of an acceptable scientific standard, however I have significant reservations, as outlined above.

## References

[1] : Policy Statement: Breastfeeding and the Use of Human Milk. *Pediatrics* .2022;150(1) : 10.1542/peds.2022-057988 10.1542/peds.2022-057988 35921640

# Response

**Published:** 2022-08-23

**Authors:** eighty mardiyan kurniawati

**Affiliations:** Airlangga University, Indonesia

Dear reviewers,

 Thank you for being willing to review our manuscript and give suggestions to improve its quality. We've tried to revise it. I hope you are satisfied with our revision.

 Thank you for your time.

 Best regards
